# Jejunal perforation caused by a feeding jejunostomy tube: a case report

**DOI:** 10.1186/1752-1947-2-224

**Published:** 2008-06-30

**Authors:** Nicholas A Stylianides, Ravindra S Date, Kishor G Pursnani, Jeremy B Ward

**Affiliations:** 1 Department of Gastrointestinal Surgery, Lancashire Teaching Hospital NHS Foundation Trust, Preston Road, Chorley, Lancashire PR7 1PP, UK

## Abstract

**Introduction:**

Percutaneous endoscopic gastrostomy and feeding jejunostomy are used for providing long-term nutritional support to patients with neurological disorders. Various mechanical complications of these procedures are described.

**Case presentation:**

We report a case of a 17-year-old boy with cerebral injury who had a percutaneous endoscopic gastrostomy tube changed to a feeding jejunostomy tube. Twenty-four hours later he developed abdominal pain and became clinically septic. A contrast study through the feeding tube and a subsequent computed tomography scan did not reveal any intra-abdominal pathology. At laparotomy it was discovered that the tip of the feeding tube had perforated through the jejunal wall and was lying outside the lumen. This was successfully treated by re-inserting a feeding jejunostomy tube distally and closure of the perforation and previous FJ site

**Conclusion:**

We suggest that the threshold for contrast studies and operative intervention should be low in neurologically impaired patients to avoid the delay in treatment of tube-related complications.

## Introduction

Percutaneous endoscopic gastrostomy (PEG) and feeding jejunostomy (FJ) are well-established methods of providing access to the gastrointestinal tract to administer enteral nutrition and medication over prolonged periods of time in patients with neurological disorders.

There is evidence to demonstrate that a FJ is a safe procedure with associated reductions of infective and metabolic complications when compared with total parenteral nutrition [1-4]. Although a relatively simple technical procedure it is not without risk or complication [5-9]. We report a rare complication secondary to insertion of a FJ.

## Case presentation

A 17-year-old boy was admitted to the surgical ward for insertion of a FJ as his PEG tube was not functioning properly. He had been involved in a road traffic accident at the age of 12 and had suffered diffuse irreversible brain injury that had left him bed-ridden and in a vegetative state. Nutritional issues had been managed successfully for 5 years by means of a PEG tube, until the 'buried bumper' of the tube made feeding difficult. The PEG tube was removed and a FJ tube was inserted via a small laparotomy, 25 cm distal to the duodenojejunal flexure.

Twenty-four hours later the patient was restless and appeared to be distressed. Clinical assessment was limited due to his inability to communicate. A contrast study was performed into the jejunum to rule out any tube-related complications; this was reported as normal. Water infusion was subsequently commenced through the FJ as per protocol.

The patient became tachycardic and pyrexial over the next 24 hours and began to vomit. His white cell count was raised at 20 × 10^9^/litre. Chest auscultation revealed right basal crepitations and a plain anterior-posterior chest X-ray showed right lower lobe consolidation. A diagnosis of aspiration pneumonia was made and he was commenced on intravenous antibiotics. The FJ was left on free drainage.

On the third postoperative day he remained septic in spite of the treatment. Clinical examination showed abdominal distension and tenderness. An urgent computed tomography scan was performed which confirmed right basal consolidation but no leak from the FJ tube and no other bowel abnormality.

**Figure 1:**
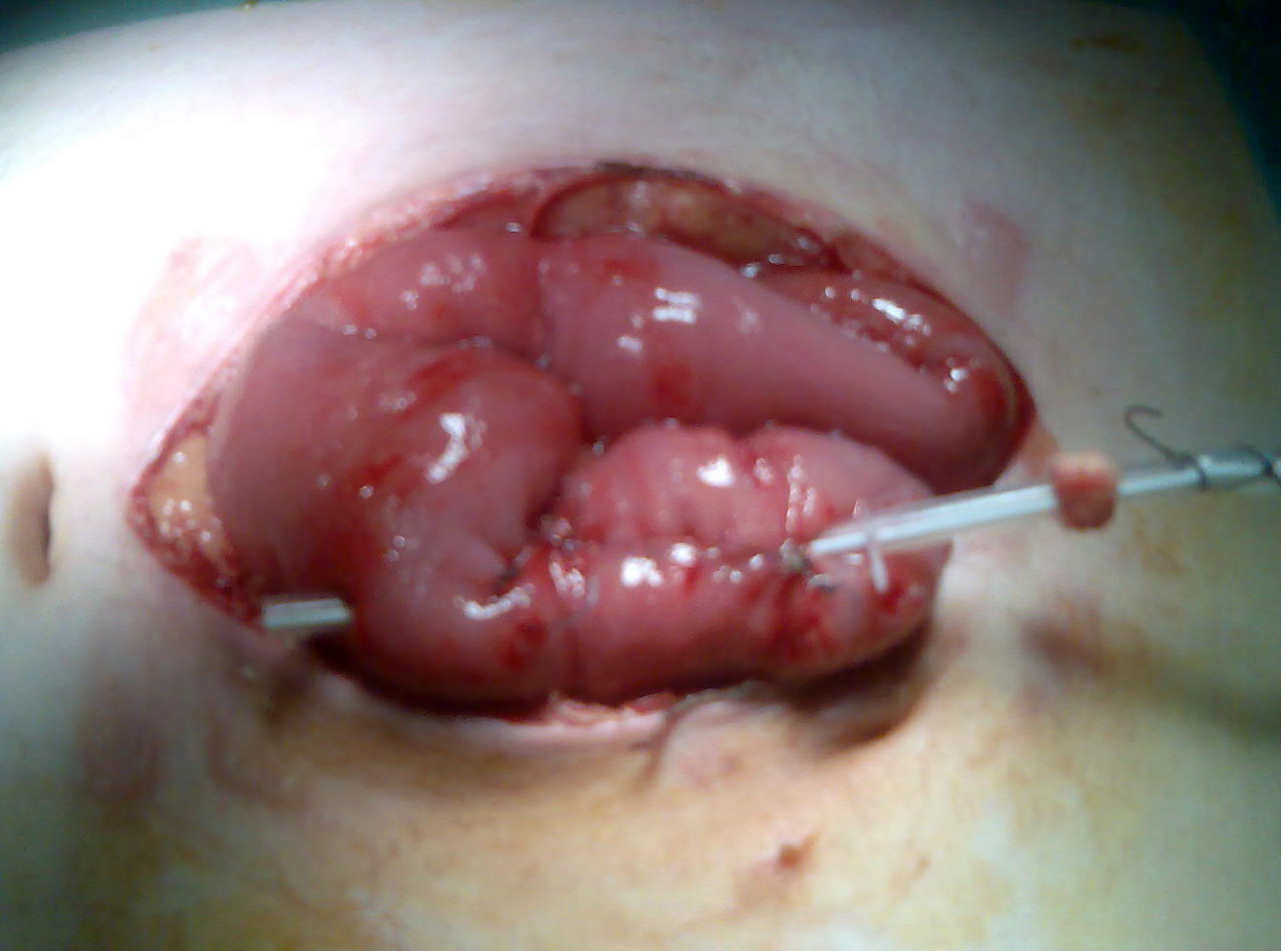
Intra-operative photograph demonstrating perforation of the jejunum by the feeding jejunostomy tube.

As his condition did not improve the decision to explore the abdomen was made on clinical grounds. At laparotomy the tip of the feeding tube was found to be lying outside the jejunal lumen having eroded directly through the wall of the small bowel (Figure 1). There was minimal spillage of bile in the peritoneum indicating recent perforation. The FJ was removed and replaced by a new tube positioned distally. The perforation and previous FJ site were closed. The patient was admitted to the intensive care unit postoperatively. He made a slow recovery and was discharged home 3 weeks postoperatively.

## Discussion

FJ is associated with high complication rates ranging between 15% and 55%. The incidence of major complications is 8% to 20%, with a jejunostomy related mortality of 2% to 10% [5].

Mechanical complications are difficult to assess clinically in neurologically impaired patients because of the lack of appropriate communication. This carries the risk of pathologies going undetected for longer periods of time with a subsequent increase in morbidity and mortality. The threshold for imaging and operative intervention should be low in such patients. Despite our repeated efforts to diagnose a tube-related complication we were unable to do so until surgical exploration. This case demonstrates the need for good clinical judgement and a high index of suspicion for tube-related complications, especially in situations where both clinical assessment of the patient and the appropriate investigations fail to provide adequate evidence of the problem.

A possible explanation for such a perforation is the presence of localised pressure necrosis of the bowel wall caused by constant pressure exerted by the tip of the feeding tube on a single point of the bowel wall. Attempts to prevent this occurring are undertaken by using appropriately designed soft-tipped tubes and by fixing the bowel wall to the anterior abdominal wall to prevent any rotation.

## Conclusion

We suggest that a low threshold for both contrast studies and operative intervention in neurologically impaired patients may be a safer way to manage feeding jejunostomy tube-related complications.

## Abbreviations

FJ: feeding jejunostomy; PEG: percutaneous endoscopic gastrostomy.

## Competing interests

The authors declare that they have no competing interests.

## Consent

Written informed consent was obtained from the patient's next-of-kin for publication of this case report and accompanying images. A copy of the written consent is available for review by the Editor-in-Chief of this journal.

## Authors' contributions

NAS helped in acquisition of data and preparation of the first draft, RSD was responsible for conception of the idea, overall preparation and revision of the manuscript, KGP and JBW were responsible for management of the patient and revising the manuscript critically for important intellectual content. All authors read and approved the final manuscript.
